# Supplementary figures and images for: Unpredictable soil conditions can affect the prevalence of a microbial symbiosis
Source: PeerJ. 2024 May 20;12:e17445. doi: 10.7717/peerj.17445 (PMC11114107; doi:10.7717/peerj.17445)

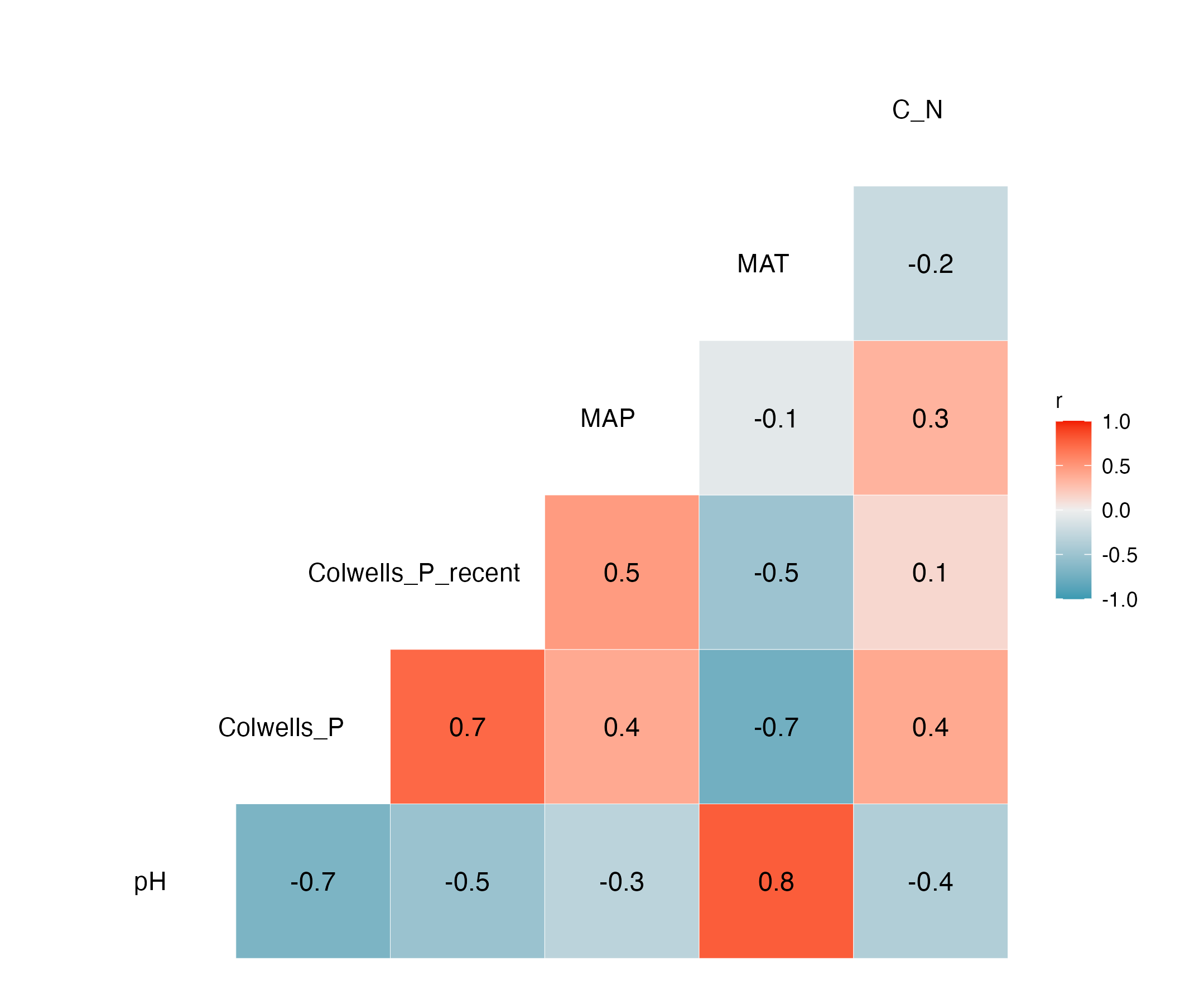

Supplement: Supplemental Information 1 [file peerj-12-17445-s001.png]

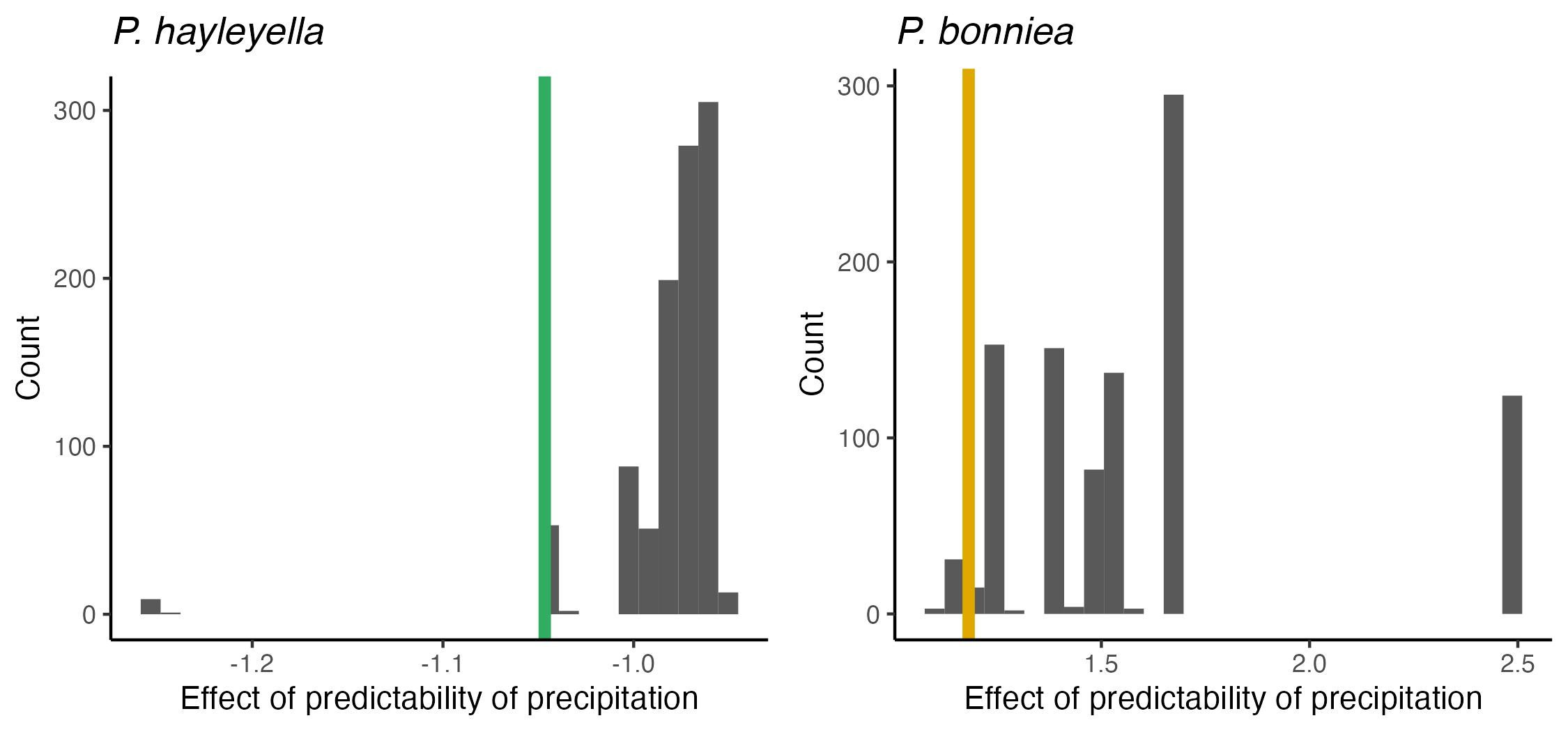

Supplement: Supplemental Information 2 — Subsamples were generated by randomly removing 350 observations from the two largest of the sampled locations. Green line shows the estimated effect of predictability of precipitation from the full model for P. hayleyella. Orange line shows the estimated effect of predictability of precipitation from the full model for P. bonniea. [file peerj-12-17445-s002.png]
